# Reduced SMAD2/3 activation independently predicts increased depth of human cutaneous squamous cell carcinoma

**DOI:** 10.18632/oncotarget.24545

**Published:** 2018-02-22

**Authors:** Aidan M. Rose, Lindsay C. Spender, Christopher Stephen, Alastair Mitchell, William Rickaby, Susan Bray, Alan T. Evans, Jasbani Dayal, Karin J. Purdie, Catherine A. Harwood, Charlotte M. Proby, Irene M. Leigh, Philip J. Coates, Gareth J. Inman

**Affiliations:** ^1^ Division of Cancer Research, School of Medicine, University of Dundee, Ninewells Hospital and Medical School, Dundee, Scotland, DD1 9SY, UK; ^2^ Department of Plastic and Reconstructive Surgery, Ninewells Hospital and Medical School, NHS Tayside, Dundee, Scotland, DD1 9SY, UK; ^3^ Department of Dermatology, Ninewells Hospital and Medical School, NHS Tayside, Dundee, Scotland, DD1 9SY, UK; ^4^ Dermatopathology Laboratory, St. John's Institute of Dermatology, St.Thomas’ Hospital, London, SE1 7EH, UK; ^5^ Tayside Tissue Bank, Ninewells Hospital and Medical School, NHS Tayside, Dundee, Scotland, DD1 9SY, UK; ^6^ Department of Pathology, Ninewells Hospital and Medical School, NHS Tayside, Dundee, Scotland, DD1 9SY, UK; ^7^ Centre for Cell Biology and Cutaneous Research, Barts and the London School of Medicine and Dentistry, Queen Mary University of London, London, E1 2AT, UK; ^8^ Regional Centre for Applied Molecular Oncology (RECAMO), Masaryk Memorial Cancer Institute, Brno, 656 53, Czech Republic

**Keywords:** TGF-β, IHC, SMAD, carcinoma, FFPE

## Abstract

The incidence of cutaneous squamous cell carcinoma (cSCC) is rising. Whilst the majority are cured surgically, aggressive metastatic cSCC carry a poor prognosis. Inactivating mutations in transforming growth factor beta (TGF-β) receptors have been identified amongst genetic drivers of sporadic tumours and murine models of cSCC, suggesting a tumour suppressor function for TGF-β in normal skin. However, paradoxically, TGF-β acts as a tumour promoter in some murine model systems. Few studies have analysed the role of TGF-β/activin signalling in human normal skin, hyper-proliferative skin disorders and cSCC. Antibodies recognising phospho-SMAD proteins which are activated during canonical TGF-β/activin signalling were validated for use in immunohistochemistry. A tissue microarray comprising FFPE lesional and perilesional tissue from human primary invasive cSCC (n=238), cSCC *in-situ* (n=2) and keratocanthoma (n=9) were analysed in comparison with tissues from normal human scalp (n=10). Phosphorylated SMAD2 and SMAD3 were detected in normal interfollicular epidermal keratinocytes and were also highly localised to inner root sheath, matrix cells and Keratin 15 positive cells. Lesional cSCC tissue had significantly reduced activated SMAD2/3 compared to perilesional tissue, consistent with a tumour suppressor role for SMAD2/3 activators in cSCC. Increased cSCC tumour thickness inversely correlated with the presence of phospho-SMADs in tumour tissue suggesting that a reduction in canonical TGF-β/activin signalling may be associated with disease progression.

## INTRODUCTION

Cutaneous squamous cell carcinoma (cSCC) is the second most common skin cancer and one of the most common human cancers worldwide [1]. Whilst most lesions are cured surgically, a small subset of tumours are associated with a high-risk of soft tissue destruction, regional metastasis and poor prognosis [2]. Given its increasing incidence defining the molecular events that drive cSCC is an urgent research need. Mutational inactivation of TGF-β receptors in skin stem cells has recently been identified as a genetic driver in a subset of cSCC, suggesting an important role for TGF-β signalling in controlling skin homeostasis [3]. This observation is consistent with roles for TGF-β signalling in the regulation of many developmental and normal cellular responses [4] including tissue homeostasis, immune responses and cell differentiation [5]. However, when the TGF-β signalling pathway is disrupted it can also contribute to various pathological states ranging from immune disorders to fibrosis and cancer [5].

During cancer development, TGF-β signalling may act as either tumour suppressor or tumour promoter in a cell-type and context dependent fashion [6]. This duality of function is readily demonstrated in murine models of skin cancer. The constitutive over-expression of TGF-β ligand appears to protect supra-basal keratinocytes from TPA-induced epidermal hyperplasia, thereby impeding tumour formation via the regulation of keratinocyte proliferation and differentiation [7]. In contrast, in the same model systems and once tumours have progressed to invasive carcinoma, TGF-β functionally switches to promote invasion and metastasis [7, 8]. Equally discrepant are the few studies to examine mediators of TGF-β signalling in normal human skin and cSCC tissue, which report contradictory findings related to the relevance and level of expression of TGF-β ligands, TGF-β receptors and SMAD transcription factors [9–11]. It remains unclear, therefore, when TGF-β signalling might act to prevent or promote tumourigenesis in this disease.

TGF-β/activin signalling can be monitored by detection of carboxyl-terminal phosphorylated forms of TGF-β receptor-regulated intracellular signalling proteins (R-SMADs). Signalling is triggered when activated ligand binds to a constitutively active TGF-β type 2 receptor (TGFBR2) at the cell membrane [12]. TGF-β:TGFBR2 complex then recruits and activates the TGF-β type 1 receptor (TGFBR1) [13] which then activates the canonical SMAD transcription factors, SMAD2 and SMAD3, via C-terminal phosphorylation (PO_4_-) at serines 465/467 and 433/435, respectively [14]. Activin signalling also induces C-terminal phosphorylation of SMAD2 and SMAD3 but via ACVR1B and ACVR2A/ACVR2B type 1 and type 2 receptors, respectively. Once phosphorylated, PO_4_-SMAD2 and PO_4_-SMAD3 form a hetero-trimeric complex with the co-operative SMAD4, and translocate to the nucleus [15]. Within the nucleus the SMAD complexes, along with other transcriptional cofactors, regulate the transcriptional activation or repression of target genes [16].

In order to better understand the role of TGF-β/activin signalling in human skin tumours (hereafter referred to as TGF-β signalling for simplicity), we characterise the expression of nuclear PO_4_-SMAD2 and PO_4_-SMAD3 as markers of endogenous canonical TGF-β signalling activity within normal human skin and primary human cSCC samples. Overall, a reduction in endogenous PO_4_-SMAD2/3 levels were identified in cSCC tumours when compared to peri-lesional skin, supporting a tumour suppressor functional role for TGF-β/activin in squamous skin cancers. Further statistical analysis of PO_4_-SMAD2/3 levels and their association with pathological features of cSCC demonstrate that increasing tumour depth (a known risk factor for nodal metastasis and poor prognosis) independently predicts a reduction in canonical TGF-β signalling activity. These findings suggest that a detectable reduction in PO_4_-SMAD2/3 levels within a primary tumour may be biologically associated with disease progression.

## RESULTS

### Validation of antibodies for IHC analysis

**Figure 1:**
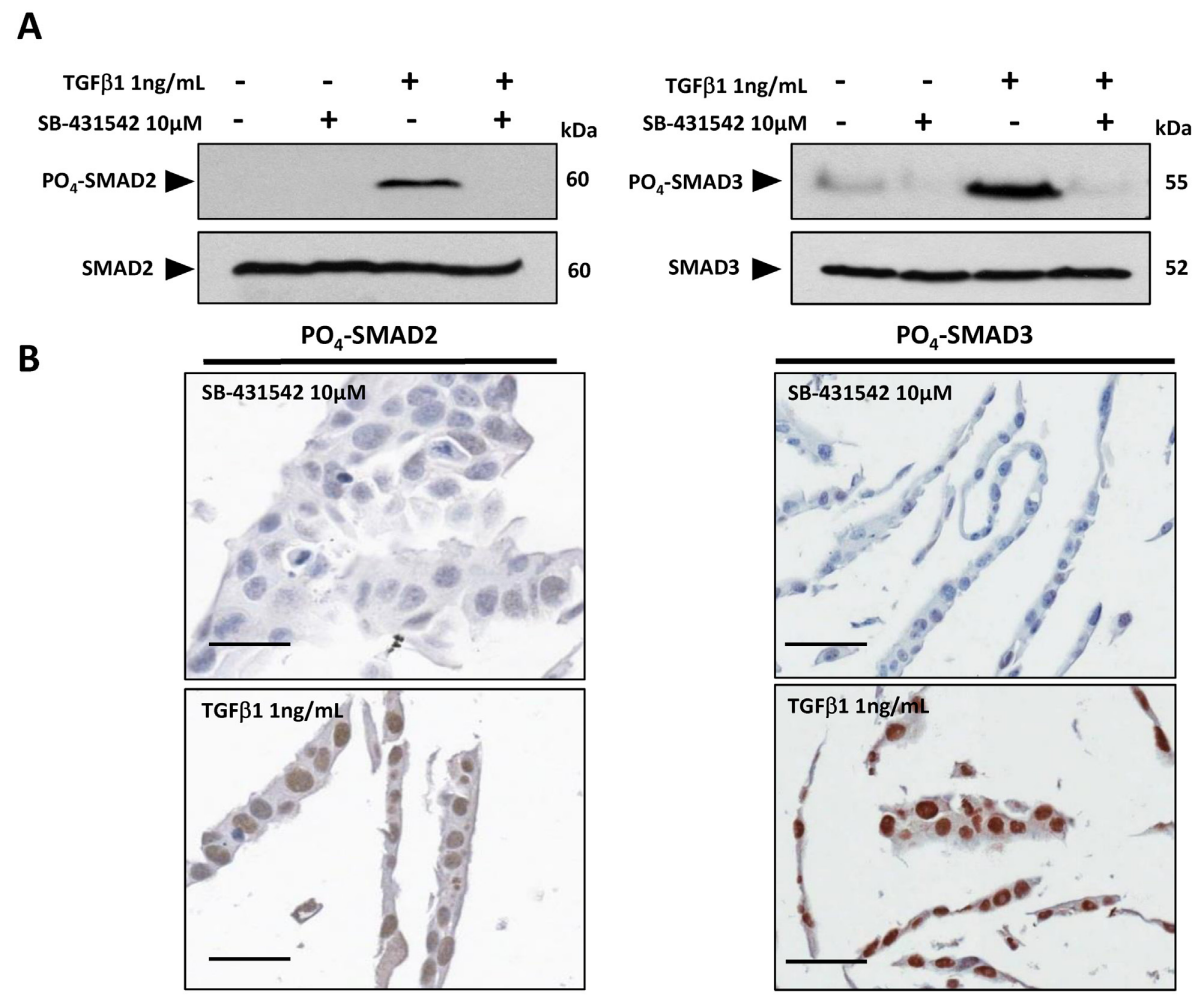
Validation of PO_4_-SMAD2 (Ser 465/467) and PO_4_-SMAD3 (Ser 433/435) antibodies for immunohistochemistry SCCIC4 cells were pre-treated with 10μM TGFBR1 (ALK5) kinase inhibitor SB-431542 or DMSO vehicle control followed by addition of either 1ng/mL TGF-β1 or vehicle control (4mM HCl) for 1 hour as indicated. **(A)** Cell lysates were analysed by SDS-PAGE and western blotting using PO_4_-SMAD2, SMAD2, PO_4_-SMAD3 and SMAD3 specific antibodies as indicated. **(B)** Parallel cell samples treated with either TGF-β1 (positive samples) or SB-431542 (negative samples) as described above were pelleted into agarose and embedded into paraffin wax for IHC. Representative digital images (x20 magnification, Aperio Imagescope) are shown. Scale bars = 50μM.

Antibodies specific for C-terminal phosphorylated forms of SMAD proteins (see supplementary methods) were validated initially for use in immunohistochemistry (IHC) on formalin-fixed paraffin embedded (FFPE) human tissue. The human cSCC cell line SCCIC4 [17] was treated *in vitro* with exogenous TGF-β1 ligand and/or the Activin-Like Kinase 4 (ALK4)/TGFBR1(ALK5)/ALK7 inhibitor, SB-431542 to induce or inhibit TGF-β signalling, respectively [18]. Western blot analysis of cell lysates confirmed active upregulation of C-terminal phosphorylation in SMAD2 and SMAD3 following treatment with TGF-β1 ligand (Figure 1A). PO_4_-SMAD2 and PO_4_-SMAD3 expression was either undetectable or reduced to below basal levels in the presence of the TGFBR1 inhibitor SB-431542 (Figure 1A). Parallel cell samples were embedded in agarose gel pellets and processed for IHC staining. FFPE SB-431542 treated SCCIC4 cells were negative for nuclear PO_4_-SMAD2 and PO_4_-SMAD3 staining. In contrast, strongly positive nuclear staining was detected in TGF-β1 treated cells (Figure 1B), validating both antibodies for IHC on FFPE human tissue.

### Endogenous TGF-β signalling activity in normal human skin

**Figure 2:**
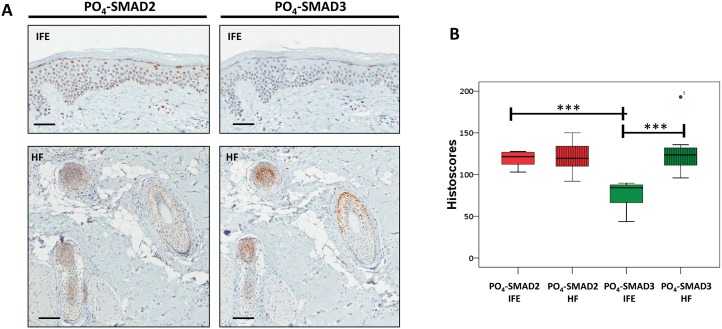
Endogenous TGF-β signalling in the interfollicular epidermis and hair follicle **(A)** Representative IHC images of normal human scalp skin stained with anti-PO_4_-SMAD antibodies as indicated. IFE = Interfollicular epidermis. HF = Transverse sections through hair follicles within dermis. **(B)** Box and whisker plots comparing IFE and HF histoscores for normal human scalp skin samples (n=10) stained with indicated antibodies. ^***^= p<0.001 (Student *t*-test). Scale bars = 200μm.

The level and distribution of PO_4_-SMAD2 and PO_4_-SMAD3 in sections from a panel of 10 normal human scalp skin samples were then analysed by IHC within two skin regions, the inter-follicular epidermis (IFE) and the hair follicles (HF). Immuno-reactivity was examined and quantified by four independent scorers using the histoscore method [19] (Supplementary Methods and Supplementary Figure 1). Positive nuclear PO_4_-SMAD2 staining was consistently identified in both IFE (mean histoscore 118 +/− 9.3 s.d.) and HF (mean histoscore 119.7 +/− 14.8 s.d) (Figure 2A-2B). Histoscores for PO_4_-SMAD3 IHC were lower in the IFE (mean histoscore 77.1 +/− 14.2 s.d) which is consistent with lower levels of total SMAD3 than total SMAD2 being detectable in isolated normal skin epidermis (Supplementary Figure 2). PO_4_-SMAD3 histoscores in adjacent HF (mean histoscore 126.8 +/− 23.8 s.d) (Figure 2A-2B) were almost double the histoscores for IFE tissue.

**Figure 3:**
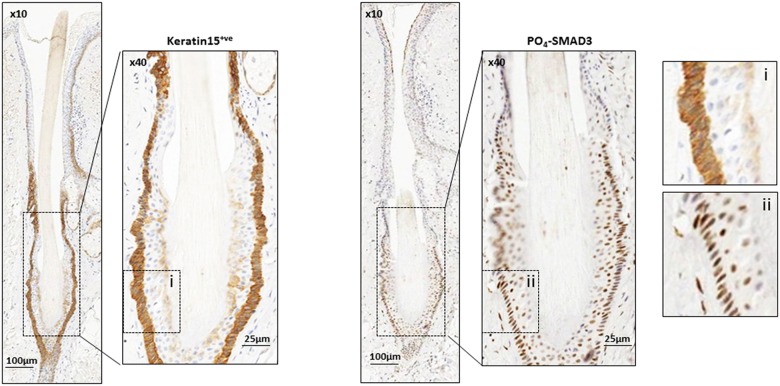
Endogenous TGF-β signalling in Keratin 15 positive hair follicle bulge stem cells Representative images of IHC of normal human scalp skin cut in serial section and stained with human bulge stem cell marker Keratin 15 (K15) and PO_4_-SMAD3, as indicated. Enlarged sections of Keratin 15 (i) and PO_4_-SMAD3 (ii) stained hair bulge are shown for comparison. Magnification and scale bars as marked.

High levels of both PO_4_-SMAD2 and PO_4_-SMAD3 immuno-reactivity were also detected in the inner root sheath and hair follicle matrix (Supplementary Figure 3A-3B). The matrix is particularly rich in highly proliferative matrix transit amplifying (MTA) cells during anagen, which are active intermediates of quiescent hair follicle bulge (HFB) stem cells [20]. We tested whether the HFB stem cells may represent a site of active endogenous TGF-β signalling. Human HFB stem cells are known to express KERATIN 15 (K15) [21] and richly populate the lower portion of the hair follicle during late catagen and telogen [22]. Serial sections of specimens, stained for both K15 and phosphorylated SMAD proteins (Figure 3), were screened specifically for late catagen and telogen hair follicles which appeared strongly positive for K15 immuno-reactivity. K15 expression consistently co-localised with PO_4_-SMAD3 expression in HF at these stages (Figure 3).

Further sites of weak endogenous TGF-β signalling within the dermis included blood vessel walls and adjacent clusters of eccrine glands (Supplementary Figure 3). These findings were confirmed by the site-specific nature of the staining and co-localisation between PO_4_-SMAD expression and the endothelial cell marker CD31 (Supplementary Figure 4A), indicating that endogenous TGF-β signalling may also be active in skin blood vessel walls. There was no correlation between PO_4_-SMAD expression and the activated fibroblast marker, α-smooth muscle actin (α-SMA) (Supplementary Figure 4B). This data indicates that in normal human epidermis, endogenous canonical TGF-β signalling exists at two sites, the IFE and the HF. Within the HF, high level activated SMAD expression appears to be present within the cellular compartments of the inner root sheath and the hair follicle matrix during anagen and K15 positive cells including the HFB stem cells during telogen stage of the hair follicle cycle.

### PO_4_-SMAD2 and PO_4_-SMAD3 levels are reduced in invasive tumours compared to perilesional tissue

**Figure 4:**
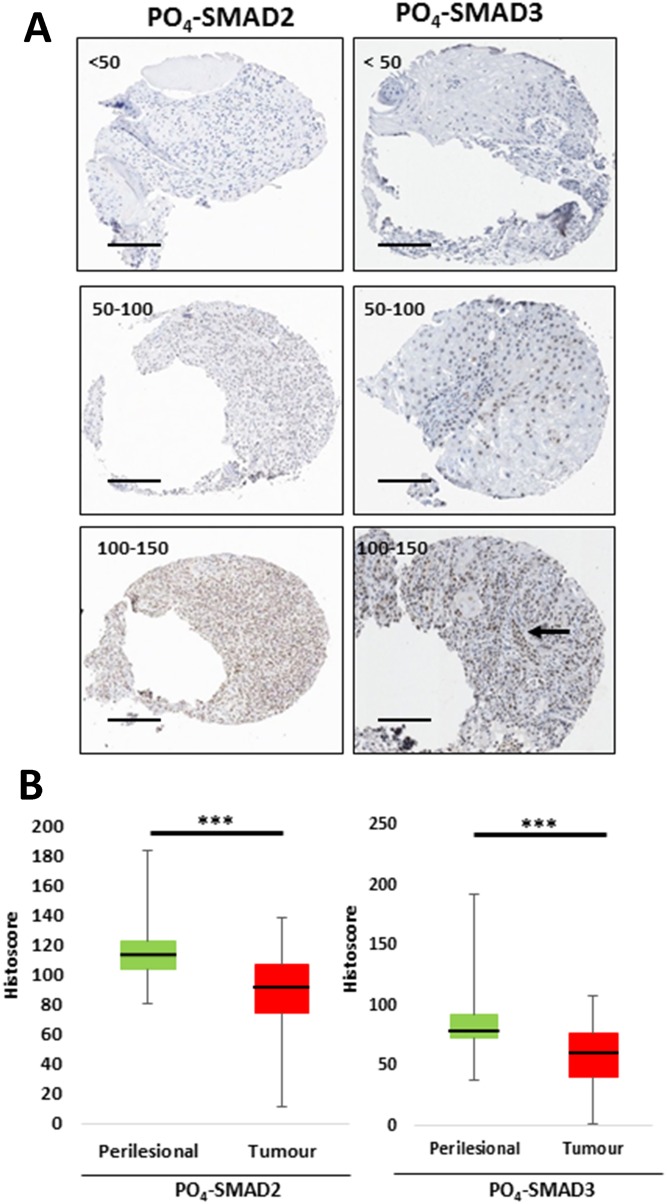
Active endogenous TGF-β signalling is significantly reduced in invasive cSCC compared to perilesional tissue **(A)** Representative images of tumour cores from TMA at 10-fold magnification, demonstrating varying degrees of histoscores from low (<50) to high (100-150) for PO_4_-SMAD2 and PO_4_-SMAD3 immunostaining as labelled. Tumour-Stroma heterogeneity labelled by arrow. **(B)** Graphical representative of mean histoscores for perilesional tissue (green) and tumour (red) across the whole TMA for PO_4_-SMAD2 and PO_4_-SMAD3 immunostaining as labelled. ^***^ p=<0.001 (Wilcoxon matched pairs test). Scale bar = 200μm.

Next, a tissue microarray (TMA) of 249 primary squamo-proliferative lesions was examined. Of the 249 samples, 230 primary cutaneous lesions were represented by more than one informative tissue core and so were included in the final histoscore analysis. Of the 230 lesions scored, 97.8% were primary cSCC (n=225) and 2.2% were KA (n=5). 77% of the lesions were excised from sun-exposed sites of the head and neck (177/230), 73.9% from male patients (170/230) and 63% from patients over 80 years old (145/230) (Table 1). The TMA was immuno-stained for PO_4_-SMAD2 and PO_4_-SMAD3. Overall, a significant reduction in active nuclear PO_4_-SMAD2 and PO_4_-SMAD3 staining was detected in invasive tumours when compared to site matched perilesional tissue (Figure 4). This finding indicates that impaired canonical TGF-β signalling activity represents a common feature of human primary invasive cSCC.

**Table 1:** Mean tumour PO_4_-SMAD histoscores for patient and tumour variables with analysis of variance

Variable	Category	Number	Percent	PO_4_-SMAD2 Tumour HISTOSCORES			PO_4_-SMAD3 Tumour HISTOSCORES		
				Mean	S.D	Sig. (ANOVA)	Mean	S.D	Sig. (ANOVA)
**Sex**	**Male**	170	73.9	89.06	24.36		57.73	23.14	
	**Female**	60	26.1	86.58	23.48	0.497	60.88	25.38	0.377
**Site**	**High-risk H&N**	66	28.7	90.33	21.92		61.83	22.98	
	**Low-risk H&N**	111	48.3	86.95	24.39		56.72	23.71	
	**Torso**	5	2.2	72.29	23.99		44.67	24.08	
	**Upper limb**	38	16.5	92.05	26.94		59.79	25.53	
	**Lower limb**	9	3.9	94.92	21.69		62.84	19.79	
	**Perineal**	1	0.4	109.13	-	0.442	29.14	-	0.645
**Grade**	**< Poorly Diff**	152	66.1	88.69	23.75		59.34	23.74	
	**≥ Poorly Diff**	78	33.9	87.88	24.94	0.81	57.01	23.75	0.481
**Tumour Depth**	**<4mm**	115	50	92.50	24.64		62.83	22.77	
	**≥4mm**	115	50	84.33	22.96	0.01	54.28	23.99	0.006
**Clark Level**	**<IV**	21	9.1	85.82	25.54		57.11	23.42	
	**≥IV**	209	90.9	88.68	24.01	0.605	58.69	23.8	0.771
**Perineural Invasion**	**Absent**	214	93	88.38	24.48		58.76	23.95	
	**Present**	16	7	88.98	18.94	0.922	55.79	21.03	0.631
**Maximum Diameter**	**<20mm**	168	73	90.81	23.36		59.97	23.58	
	**≥20mm**	62	27	81.92	25.09	0.013	54.7	23.88	0.135
**Depth of Invasion**	**Up to subcutaneous fat**	205	89.1	89.01	24.29		60.2	23.2	
	**Beyond subcutaneous fat**	25	10.9	83.5	22.41	0.282	45	24.07	0.002

S.D = Standard Deviation, H&N = Sun-exposed head and neck, Clark Level IV = Invasion into the reticular dermis, ANOVA = One-way analysis of variance (significant *p*-value = <0.05).

Clinical and pathological variables pertinent to cSCC tumour staging [23] were then examined for association with PO_4_-SMAD2 or PO_4_-SMAD3 histoscores (Table 1 and Supplementary Figures 5-6). Statistical analysis of variance (ANOVA; significant values *p*=< 0.05) revealed no significant differences in mean PO_4_-SMAD2 or PO_4_-SMAD3 histoscores analysed by patient sex, patient age, tumour pathological type (Table 1), tumour grade (Table 1 and Supplementary Figure 6A), tumour site (Table 1 and Supplementary Figure 5), the presence of peri-neural invasion (PNI) (Table 1 and Supplementary Figure 6B) or Clark level of invasion (Table 1 and Supplementary Figure 6C).

**Figure 5:**
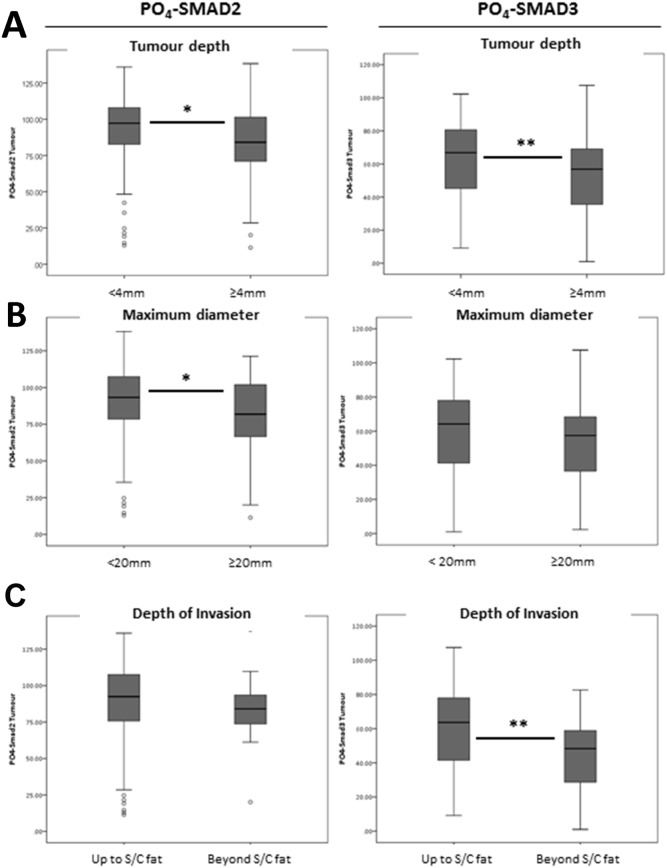
TMA histoscores identify a significant association between mean nuclear R-Smad expression and cSCC tumour depth, diameter and pathological depth of invasion **(A)** Nuclear PO_4_-SMAD2 and PO_4_-SMAD3 activity by tumour depth, defined as <4mm and ≥4mm. **(B)** Nuclear PO_4_-SMAD2 and PO_4_-SMAD3 activity by maximum tumour diameter, defined as <20mm or ≥20mm. **(C)** Nuclear PO_4_-SMAD2 and PO_4_-SMAD3 activity by pathological depth of invasion. Grouped as invasion up to subcutaneous fat and invasion beyond subcutaneous fat. Analysis of Variance (ANOVA) Sig ^*^ *p* = <0.05, ^**^ p = <0.01.

There was a significant reduction in mean PO_4_-SMAD2 histoscores seen in larger diameter tumours (≥20mm versus <20mm) (Table 1 and Figure 5B, ANOVA; *p*=0.013) and a significant reduction in mean PO_4_-SMAD3 histoscores seen in tumours invading beyond subcutaneous fat (Table 1 and Figure 5C, ANOVA; *p*=0.002). There were also significant reductions in mean PO_4_-SMAD2 and PO_4_-SMAD3 histoscores detected in thick tumours (≥4mm versus <4mm) (Table 1 and Figure 5A, ANOVA; *p*=0.01 and p=0.006, respectively).

### Reduced PO_4_-SMAD2 and PO_4_-SMAD3 levels correlate with large and thick invasive tumours

To investigate in more detail the potential association between PO_4_-SMAD histoscores and high-risk cSCC (defined as Breslow tumour depth ≥4mm, tumour diameter ≥20mm and invasion beyond subcutaneous fat), the data was re-analysed for significant correlations using Spearmann's rank correlation co-efficient (C.C) (Table 2). High-risk tumour depths (≥4mm) demonstrated a highly significant negative dependence on both PO_4_-SMAD2 (Table 2, C.C −0.214; p=0.001) and PO_4_-SMAD3 (Table 2, C.C −0.200; p=0.002) (Supplementary Figures 7A and 8) histoscores, suggesting a correlation between thicker tumours and lower SMAD2/3 phosphorylation. A subtle but significant negative dependence was also detected between higher maximum diameter tumours and PO_4_-SMAD2 histoscores (Table 2, C.C −0.167; p=0.011, Supplementary Figure 7B). Consolidating these findings, lower levels of PO_4_-SMAD3 correlated with tumour invasion beyond the depth of subcutaneous fat (Table 2, C.C −0.187; p=0.005, Supplementary Figure 7C).

**Table 2:** Statistical correlations between cSCC perilesional and tumour TGF-β activity and selected “High-Risk” cSCC pathological variables

			PO_4_-SMAD2	PO_4_-SMAD3
**Spearman's**	**PO_4_-SMAD2**	C.C		.366^***^
		Sig. (2-tailed)	.	**.000**
	**PO_4_-SMAD3**	C.C	.366^***^	
		Sig. (2-tailed)	**.000**	.
	**Depth >4mm**	C.C	-.214^**^	-.200^**^
		Sig. (2-tailed)	**.001**	**.002**
	**Diameter >20mm**	C.C	-.167^*^	-.115
		Sig. (2-tailed)	**.011**	.081
	**Beyond subcutaneous fat**	C.C	-.101	-.187^**^
		Sig. (2-tailed)	.125	**.005**

C.C = Correlation co-efficient, Sig. = Significance, N = Number, S/C = Subcutaneous.

^***^Correlation is significant at p = <0.001 level (2-tailed). ^**^Correlation is significant at p = < 0.01 level (2-tailed). ^*^Correlation is significant at p = < 0.05 level (2-tailed).

### Higher tumour depth independently predicts lower endogenous TGF-β signalling activity

Next, a binary logistic regression analysis was performed, using the binary outcome PO_4_-SMAD2/PO_4_-SMAD3 “off” (negative) (=1) and PO_4_-SMAD2/PO_4_-SMAD3 “on” (positive) (=0) as the dependent variable (Table 3). The cut-off used for detecting PO_4_-SMAD2/PO_4_-SMAD3 “off” was determined as a tumour histoscore of less than the 25^th^ percentile of the mean in both PO_4_-SMAD2 and PO_4_-SMAD3 staining. This analysis identified tumour depth as the only significant independent predictor of low PO_4_-SMAD expression (Table 3, Wald (*Chi-squared*) = 7.447; p = 0.006; Exp(B) = 1.127, 95% C.I 1.034-1.227), indicating that for each unit increase in tumour depth (mm), the odds of a tumour expressing a histoscore (for both PO_4_-SMAD2 and PO_4_-SMAD3) at levels below the 25^th^ percentile of the mean increased by 12.7% (Table 3, (1.127^*^100)-100) – with a 95% C.I of 3.4%–22.7%). This analysis further strengthens the hypothesis that TGF-β signalling is likely to be acting primarily as a tumour suppressor in human primary cSCC and that impairment in canonical TGF-β signalling may be advantageous for tumour cell growth and invasion.

**Table 3:** Binary logistic regression model identifies tumour depth as an independent predictor of lower cSCC R-Smad activity

Variable	DF	Χ^2^ (Wald)	P (<0.05)	R.R	95% CI
Depth	1	7.447	0.006	1.127	1.034-1.227
Poorly diff	1	3.125	0.077		
Site = Lower limb	1	1.168	0.280		
Diameter	-	1.107	0.293		
Invasion beyond fat	1	0.739	0.390		
Site = Torso	1	0.629	0.428		
Clark level ≥IV	1	0.140	0.708		
Site = Upper limb	1	0.114	0.736		
Site = High-risk H&N	1	0.82	0.774		
PNI	1	0.48	0.827		
Site = Low-risk H&N	1	0.019	0.889		

Binary outcome = TGF-β Signalling “off” ^*^ (0=No, 1=Yes).

^*^≤25^th^ Percentile of the mean (PO_4_-SMAD2 = ≤ 76, PO_4_-SMAD3 = ≤ 41). B = Co-efficients, S.E = Standard error of co-efficients, Wald = Wald chi-squared value. df = degree of freedom, Sig. = 2-tailed *p*-value for testing null hypothesis. Exp(B) = Exponentiation of co-efficients (Odds ratios), C.I = Confidence intervals. H&N = Sun-exposed head and neck, Clark Level IV = Invasion into the reticular dermis, PNI = Perineural invasion.

## DISCUSSION

### Detecting endogenous TGF-β activity in normal human skin

TGF-β plays vital homeostatic roles in normal skin and during skin injury repair, but some data from models of skin carcinogenesis are contradictory. The ligand appears to be consistently expressed throughout normal human epidermis [9, 11, 24] and its constitutive over-expression protects murine supra-basal keratinocytes from DMBA/TPA-induced epidermal hyperplasia [7, 25]. This protection appears to coincide with the induction of TGF-β's obligate type 2 receptor (TGFBR2), indicating a vital role for TGF-β signalling in skin homeostasis. TGFBR2 is also strongly expressed in normal human IFE [9, 11]. However, expression of active TGF-β in the basal layer is also associated with inflammation and hyper-proliferation [26].

Investigating the role of TGF-β superfamily ligands in skin homeostasis and disease has been problematic because of a lack of suitable reagents. Some researchers have relied on the detection of the TGF-β latency-associated peptide, produced as part of the TGF-β latent precursor, as a surrogate marker of TGF-β ligand expression in tissues [7]. Since, in this form, TGF-β ligand is an inactive precursor protein, the biological relevance of IHC detection of ligand, associated peptides or receptor levels as tumour markers of endogenous signalling activity is debatable [27]. The formation of ligand-receptor complex is known to drive rapid depletion of receptors from the cell surface, which rate-limits the activation of down-stream TGF-β signalling [28]. Thus, in this study, neither the expression of TGF-β superfamily ligands or TGFBR2 were assessed because commercially available antibodies could not be validated for IHC on FFPE tissue to the same rigorous validation standards that we achieved for the anti- PO_4_-SMAD2 and PO_4_-SMAD3 antibodies. Nuclear PO_4_-SMAD2 expression was consistently observed within the human epidermis in this study and in others [9, 11, 29]. C-terminal phosphorylation of the R-SMAD's is known to be sufficient for their nuclear accumulation and transcriptional activity [4]. As such, levels of phosphorylated R-SMAD's can be correlated directly with TGF-β signal strength and transcriptional responses [30]. The selection of nuclear PO_4_-SMAD2/3 expression was therefore considered appropriate as a direct marker of endogenous TGF-β signalling activity in human skin. At present though, we cannot formally identify which ligand has potential tumour suppressor function since other TGF-β superfamily members can also activate canonical PO_4_-SMAD2 and 3 via their own distinct serine threonine receptor kinase complexes including Activin, Nodal, and GDF-8 (myostatin) [31]. Activins are of particular interest, as they appear to share many of the regulatory characteristics of TGF-β in keratinocytes [32]. Activin ligands are expressed mainly in the dermis, but their obligatory type 1 receptor (ACVR1B) is expressed in the epidermis, indicating that they could act in a paracrine fashion to regulate keratinocyte proliferation [33]. Co-incidentally, Activin-A preferentially induces PO_4_-SMAD3 expression in HaCaT human keratinocyte cells [34], thus other TGF-β superfamily members may also contribute to SMAD activation in human skin.

In keeping with TGF-β-mediated regulation of keratinocyte proliferation and differentiation, the level of nuclear PO_4_-SMAD2 expression was consistently high within the IFE, but, IFE PO_4_-SMAD3 histoscores were significantly lower overall. This apparent discrepancy could reflect a differential expression of total SMAD2 and SMAD3 proteins within the IFE. We could not assess total protein levels of non-phosphorylated SMADs in FFPE sections as, again, the specificity of these antibodies could not be confirmed and there is a paucity of published data on total SMAD2 or SMAD3 protein expression in normal human skin samples. A single study described expression of total SMAD2 and SMAD3 in the upper epidermal layers and hair follicles, but reported no obvious differences in levels [35]. However, our analysis of isolated epidermis from four normal skin donors, suggests that the levels of total detectable SMAD3 are lower than SMAD2 which could account for the differential detection of phosphorylated SMAD proteins seen in this study. It is also possible that there is differential activation (PO_4_-) of SMAD proteins within the epidermis but little is known about the potential biological mechanisms, or the affects, of any selective activation of SMAD2 versus SMAD3 [36].

Immunostaining of human HF's suggests that both SMAD2- and SMAD3-dependent TGF-β signalling are active in these specialised structures. Interestingly, this active signalling was detected at seemingly dichotomous points in the hair follicle cycle, localised to the proliferative MTA cells of anagen (growth phase) follicles, but also the Keratin 15 positive HFB stem cells of telogen (resting phase) follicles. Endogenous TGF-β1 is known to suppress proliferation and induce apoptosis of MTA cells of the hair follicle bulb, driving the transition from anagen to catagen phases of the hair follicle cycle [37, 38]. This transition is characterised by a decline in proliferation and the differentiation of MTA cells in the hair follicle bulb. The MTA cells appeared rich in PO_4_-SMAD2 and PO_4_-SMAD3 expression in this study, suggesting that endogenous SMAD-dependent TGF-β1-driven signalling may be an integral regulator of human MTA cells. The expression of phosphorylated R-SMADs was also consistently localised to the HFB stem cells during telogen, consistent with recent murine studies [39]. This expression appears to be driven by the secretion of TGF-β2 ligand from adjacent DP cells, which in turn negatively regulates inhibitory BMP signalling, resulting in the activation of telogen HFB stem cells which drive hair follicle regeneration and a transition from telogen to anagen [39]. Our data are consistent with similar regulatory functions of TGF-β in human hair follicles.

### TGF-β signalling in human cSCC

High-throughput genomic analyses of sporadic human invasive cSCC have demonstrated the presence of frequent mutations in TGF-β receptors [3, 40, 41], indicating that TGF-β signalling is likely to act as a key tumour suppressor, and that mutational inactivation of this pathway may be a key driving event in tumour formation [3]. This proposed role is supported by evidence from three human “models” of squamous skin tumours: a clear genotype-phenotype correlation between germline inactivating *TGFBR1* mutations and families suffering from multiple self-healing squamous epithelioma (MSSE) [42], the presence of *TGFBR1* mutations in sorafenib-induced skin tumours [43] and clinical cancer trial data reporting spontaneous cSCC arising as a side-effect of systemic treatment with the pan-TGF-β ligand antibody, GC1008 [44].

Our current study, reporting an analysis of possibly the largest published TMA of primary human cSCC to date, supports the idea that loss of canonical TGFβ pathway signalling is associated with cSCC disease progression. Overall, despite some contradictory evidence, data from alternative IHC studies in human disease appear consistent with our finding of a trend towards attenuation of TGF-β signalling [9–11, 35]. Whilst Harradine *et al* reported elevated SMAD activation, their analysis discussed only a direct comparison of mediators of TGF-β signalling between tumours from organ transplant recipients and sporadic tumours from the non-transplant recipient population [11]. The same data, when comparing tumour progression from normal to invasive carcinoma, irrespective of tumour host, suggests an overall reduction in PO_4_-SMAD2 staining intensities [11]. This current study provides the added assurances that we compare lesional with peri-lesional uninvolved tissue and that the antibodies used were carefully validated prior to use.

The histoscore scoring method averages variation in immunostaining across tissue cores, therefore, the extent of possible heterogeneity of TGF-β signalling throughout the tumour tissue was not directly addressed in this study. A more in-depth examination of cell or site-specific TGF-β signalling activity in cSCC (e.g active signalling localised to the leading invasive edges of tumour) might reveal further clues as to where the likely biological roles for active TGF-β signalling lie.

No previous study has examined correlations between known “high-risk” pathological features of cSCC tumours and the degree of active TGF-β family signalling. A consistent association and significant correlation between low PO_4_-SMAD2/3 expression and thick tumours (≥4mm depth) was identified. Importantly, the strength of this association was such that increasing tumour depth remained an independent predictor of low tumour PO_4_-SMAD expression when accounting for all other “high-risk” variables. This correlation provides further evidence that TGF-β signalling appears to be acting primarily as a tumour suppressor in cSCC and suggests that a reduction in active TGF-β signalling may provide a significant growth advantage for affected tumour cells - as recently demonstrated *in-vitro* using primary human cSCC cell lines carrying mutant TGFBR2 and lacking canonical signalling potential [3]. As tumour thickness represents a pathological marker of invasion, it also indicates that active canonical SMAD signalling may not preclude features of tumour progression, such as tumour invasion, in human cSCC.

Recent studies in mice suggest that HF and IFE stem cells are epigenetically primed differentially to undergo epithelial to mesenchymal transition during tumourigenesis, with HF derived cells more likely to acquire a canonical TGF-β/SMAD2-driven EMT phenotype than IFE derived cells [45]. Thus, the epigenetic state of the cancer cells of origin may have a significant impact on the outcome of TGF-β mediated signalling. Since EMT is reported to be required for SCC dissemination [46], how these observations relate to tumourigenic events in human skin warrants further investigation. This study indicates that reduced canonical signalling via the SMADs correlates with high risk phenotypic changes, however, as the TGF-β pathway can also activate non-SMAD pathways such as the Mitogen Activated Protein (MAP) kinase pathway, Rho-like GTPase pathways (RhoA) and Phosphatidylinositol-3-kinase (PI3K)/Protein Kinase B (AKT) pathways [47], it is possible that, as TGF-β's SMAD-dependent tumour suppressor arm is impaired, pro-tumourigenic non-SMAD pathways dominate to drive invasion and metastasis [48]. Interestingly, the detection of EMT markers in SCC tumours correlates with upregulation of phosphorylated forms of active AKT, suggesting that signalling through AKT may be involved in EMT and could be targeted to suppress invasion [46]. It is unknown, however, whether AKT activation in SCC is TGF-β receptor dependent. Further analysis of the TMA for expression of both SMAD-dependent and non-SMAD dependent target genes and their correlation with PO_4_-SMAD expression may shed further light on whether such cross-talk has a role in human cSCC tumour tissue.

Significant tumour thickness (or depth of invasion) is a known risk factor for nodal metastasis (NM) in human cSCC [49, 50]. Our findings lead to the hypothesis that a reduction in signalling via SMADs may also correlate with increased risk of NM. This was not tested directly as the number of patients re-presenting with NM was too low to justify the analysis at this current time (n=2/249; data not shown). Such analysis was also compounded by two patient factors: the first being that most patients presenting with cSCC were elderly (63% over 80 yrs old in this study) which limits possible follow-up times and the likelihood of disease recurrence prior to mortality unrelated to cSCC disease progression; and secondly, the fact that the majority of the patients in this study presented with SCC between 2012 and 2014 and, to date, the follow-up times are too short to accumulate sufficient numbers of NM for analysis. There is an established link between loss of TGF-β signalling and the mode of tumour invasion, for example, murine and rat *in-vivo* breast carcinoma models reveal that blockade of TGF-β signalling drives cohesive (or collective) tumour cell migration and invasion [51, 52]. Importantly, cells invading collectively appear primed for lymphatic rather than blood-borne spread [51] in both breast cancer and HNSCC models [52, 53]. If this process could also be demonstrated in the context of cSCC, it would suggest that a reduction in active TGF-β signalling could prime cSCC tumour cells for collective tumour cell invasion and potentially drive their natural predilection for lymphatic spread. Given this, further investigation into the loss of endogenous PO_4_-SMAD activity as a biomarker for aggressive disease in cSCC is warranted.

## MATERIALS AND METHODS

This study was conducted according to the Declaration of Helsinki Principles. Ethical approval was obtained from the local NHS Tayside Research Ethics Committee - Research Ethics Number: NHS REC 08.S1401.69 – “The Molecular Pathogenesis of Non-melanoma Skin Cancers”. All patients participating in this study provided written, informed consent. Normal scalp skin was only obtained as redundant “dog-ear” tissue following minor plastic surgery procedures for benign lesions. Lesional and peri-lesional tissue from a total of 249 patients presenting to the Ninewells Hospital Plastic Surgery and Dermatology Departments, between 2007 and 2013, were included in this study (see Table 1 for patient/lesion details). In total, 249 sporadic primary squamo-proliferative skin lesions from immunocompetent patients (one tumour per patient) were identified and included in the array, including: 238 primary invasive cSCC, 2 cSCC *in-situ* and 9 keratoacanthoma. Of these samples, 230 were represented by more than one independent tissue core on the TMA (92%). Of these 230 tumours, 150 (65%) had more than one sufficient adjacent peri-lesional skin core for analysis.

### Optimisation of immunohistochemistry (IHC) conditions

Primary human cSCC cell lines, SCCIC4 and SCCIC18 were cultured in standard conditions using RM^+^ media (10% FCS) as previously described [54]. Adherent sub-confluent monolayers of at least 5×10^6^ cells in 75 cm^2^ tissue culture flasks were treated with either vehicle control (4 mM HCl, 1 mg/mL BSA; DMSO, 1:1000 dilution), 1 ng/mL TGF-β1 (positive sample) or 10 μM TGFBR1 kinase inhibitor SB-431542 (negative sample) [18] for 1 hour. Cells were fixed in formalin, the cell pellets re-suspended in agarose and then embedded in paraffin prior to sectioning and IHC staining as previously described [55].

### Western blotting

Cells were lysed in SDS lysis buffer and analysed by SDS-polyacrylamide gel electrophoresis and western blotting. Bound immunocomplexes were detected by enhanced chemiluminescence (ECL; Amersham). All primary and secondary antibodies used are described in supplementary information.

### IHC of FFPE tissue sections

Normal skin samples were fixed immediately in 4% paraformaldehyde and processed using a standard automated histology processor prior to embedding into paraffin wax blocks. For all IHC staining four-micron thick sections were cut onto Polysine-coated slides and air dried at room temperature prior to de-paraffinisation and rehydration via a graded alcohol series. Citrate buffer antigen retrieval and DAKO autostaining (Ely, Cambridgeshire, UK) using Vectastain^®^ ABC kits (Vector Labs, Burlingame, CA, USA) was performed as per manufacturer's instructions and as described in supplementary methods. The antibodies used for IHC are described in supplementary methods.

### Tissue microarray (TMA)

Lesions were identified by the retrospective collection of pathology reports containing minimum datasets for primary cSCC [56]. H&E slides were then reviewed, marked and used as templates for coring from their corresponding paraffin blocks using a manual tissue arrayer. A maximum of six x 1mm cores at least 3mm thick per specimen were targeted for inclusion. Cores were constructed in a grid design, facilitated by TMA Designer^R^2 software. Matched lesional and peri-lesional samples were arranged in random order.

### Image processing and digital image analysis

All stained sections were scanned using the Aperio® Scanscope XT slide scanner (Aperio® Technologies, Inc., Vista, CA) and visualised digitally online using the Aperio e-slide manager via the Tayside Tissue Bank: https://aperio-sql.tissuebank.dundee.ac.uk/

### Quantification of immunostaining and statistical analyses

All tissue sections were scored for protein expression levels via a simplified Histoscore method [19] as described in Supplementary methods. To account for heterogeneity of staining between tumour and stroma, only positive tumour cells and skin keratinocytes were scored. Scoring was undertaken remotely by four trained independent investigators blinded to the TMA structure and original pathology reports. Statistical analysis included paired samples *T*-test (Sig. p<0.05), non-parametric correlations between staining intensities and clinic-pathological variables (Spearmans-Rho - Sig. p<0.05) and binary logistic regression. All statistics were performed using SPSS v20 (IBM).

## SUPPLEMENTARY MATERIALS FIGURES AND TABLES



## Abbreviations

cSCC: cutaneous squamous cell carcinoma
IHC: immunohistochemistry
FFPE: formalin-fixed paraffin embedded
TGF-β: transforming growth factor beta
MTA: matrix transit amplifying
TMA: tissue microarray
IFE: inter-follicular epidermis
HF: hair follicles
ALK: activinlike kinase.
