# Supplementary material for: Reduced SMAD2/3 activation independently predicts increased depth of human cutaneous squamous cell carcinoma
Source: Oncotarget. 2018 Feb 22;9(18):14552–66. doi: 10.18632/oncotarget.24545 (PMC5865689; doi:10.18632/oncotarget.24545)
Supplement: Supplementary file 1 [file oncotarget-09-14552-s001.pdf]

Reduced SMAD2/3 activation independently predicts increased depth of human cutaneous squamous cell carcinoma

SUPPLEMENTARY METHODS

Immunostaining

Antigen retrieval was performed using a standard microwave based method using a total of 500mL of 10mM Citric Acid buffer (pH 6.0). Sections were boiled in a pressure cooker for 10 minutes before being immunostained on a DAKO autostainer (Ely, Cambridgeshire, UK) using Vectastain®ABC kits (Vector Labs, Burlingame, CA, USA) according to the manufacturer's instructions. Briefly, sections were blocked in normal goat serum containing 10% (v/v) from stock avidin solution (Vector Labs) for at least 20 minutes. The required concentration of primary antibody was prepared in blocking buffer containing 5% Biotin (Avidin/Biotin Blocking Kit; Vector Labs). Prior to treatment with primary antibody slides were rinsed in PBS and then left to incubate in a humid tray overnight at 4-6°c. Secondary biotinylated antibody and peroxidase ABC detection reagents (Vector Elite ABC Peroxidase Kits) were prepared: Secondary antibody as a 1/250 dilution in blocking buffer and the Avidin-Biotin-Complex (ABC) as a 1/50 dilution of reagent A (Avidin) and reagent B (Biotinylated peroxidase) in PBS. Excess PBS was removed from washed slides prior to direct application of secondary antibody and incubation at room temperature for 30 minutes. Pre-made ABC was then pipetted directly over the sections prior to incubation at room temperature for 30 minutes. Following ABC treatment, excess PBS was removed and replaced with liquid 3,3′-Diaminobenzidine (DAB) solution (Sigma-Aldrich) for approximately 10 minutes prior to rapid washes in cold tap water and counterstaining with Mayer's haematoxylin (Sigma-Aldrich) for 1 min. Finally, sections are washed again in water prior to a further reverse series of graded ethanol baths, isopropyl alcohol and HistoClear and mounting with coverslips over DPX (Distrene, Plasticiser, Xylene) mounting medium.

Quantification of immune-reactivity

The Histoscore method is a weighted scoring system analysing both staining intensity and the percentage of cells stained at that intensity (Nenutil et al., 2005). It is calculated by the following equation: (0 × % cells staining negative) + (1 × % cells staining weakly positive) + (2 × % cells staining moderately positive) + (3 × % cells staining strongly positive). There is a maximum score of 300 (100% cells staining strongly positive) and a minimum score of 0 (100% cells negative).

Protein analysis of normal human keratinocytes and epidermal skin

Normal human keratinocytes (NHK) were isolated and cultured as described previously [1]. For protein isolation, 3×10^6 NHKs along with 2×10^5 3T3 feeder cells were seeded in a 60mm dish and grown to confluence. Once NHKs were confluent all contaminating 3T3 feeders were removed using a diluted trypsin solution (1:3, trypsin: PBS) and protein was isolated from NHK's using the SDS lysis buffer. Epidermis from four different donors was separated from frozen skin using a scalpel.

Primary antibodies for western blotting

Antigen	Source	Species and Specificity	Epitope	Molecular Weight (kDa)	Concentration
**PO_4-SMAD2**	3108, Cell Signaling	Rabbit; Monoclonal	PO_4-Ser465/467	60	1:500
**PO_4-SMAD3**	52903, Abcam	Rabbit; Monoclonal	PO_4-Ser423/425	55	1:1000
**SMAD2**	3122, Cell Signalling	Rabbit; Monoclonal	Trp85	60	1:1000
**SMAD3**	9523, Cell Signalling	Rabbit; Monoclonal	Residues amino terminus	52	1:1000
**β-Actin**	A2228, Sigma	Mouse Monoclonal	N-terminal peptide	42	1:10000

Secondary antibodies for western blotting

Target	Source	Species and Specificity	Concentration
**Mouse IgG**	P0448, Dako	Goat;Polyclonal	1:2000
**Rabbit IgG**	P0260, Dako	Goat;Polyclonal	1:2000

Frozen pieces of epidermal skin were powdered using a pre-cooled mortar and pestle on dry ice. SDS lysis buffer was added before allowing the epidermal skin pieces to defrost. Immunoblotting was performed to assess the levels of total SMAD2 (Cell signalling technology, 3103, 1:500) and SMAD3 (Cell signalling technology, C67H9, 1:1000) in the four individual protein lysates. β-Actin was used as a loading control (Sigma, A2228, 1:10000).

REFERENCE

1. Watt SA, Pourreyron C, Purdie K, Hogan C, Cole CL, Foster N, Pratt N, Bourdon JC, Appleyard V, Murray K, Thompson AM, Mao X, Mein C, et al. Integrative mRNA profiling comparing cultured primary cells with clinical samples reveals PLK1 and C20orf20 as therapeutic targets in cutaneous squamous cell carcinoma. Oncogene. 2011; 30:4666–77.

Antibodies for IHC

Antigen	Category Number and Source	Species and Specificity	Epitope	Antigen Retrieval	Conc.
**PO_4-SMAD2**	3108, Cell Signaling	Rabbit; Monoclonal	PO_4 Ser465/467	Citrate Buffer	1:800
**PO_4-SMAD3**	52903, Abcam	Rabbit; Monoclonal	PO_4 Ser423 /425	Citrate Buffer	1:500
**αSMA**	M0851 (Clone 1A4), Dako	Mouse; Monoclonal	N-terminal	Citrate Buffer	1:5000
**CD31 (PECAM-1)**	04-1074, Merck Millipore	Rabbit; Monoclonal	Residues on PECAM-1	Citrate Buffer	1:40000

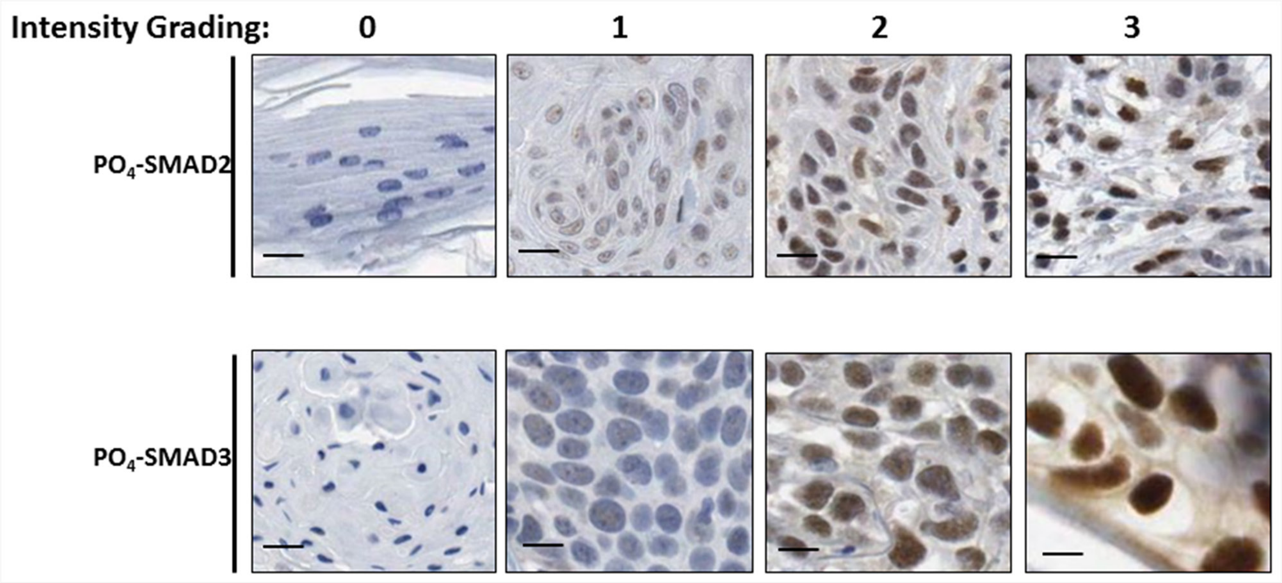

Supplementary Figure 1: Range of IHC staining intensities for nuclear PO_4-SMAD2 and PO_4-SMAD3 antibodies used for histoscore analysis of human tissue. The intensity of immuno-reactivities in skin and cSCC samples stained with indicated antibodies were interrogated by histoscore using the above guides: 0 = minimum intensity to 3 = maximum intensity. Images of nuclear staining at x20 magnification. Scale Bars = 10µm.

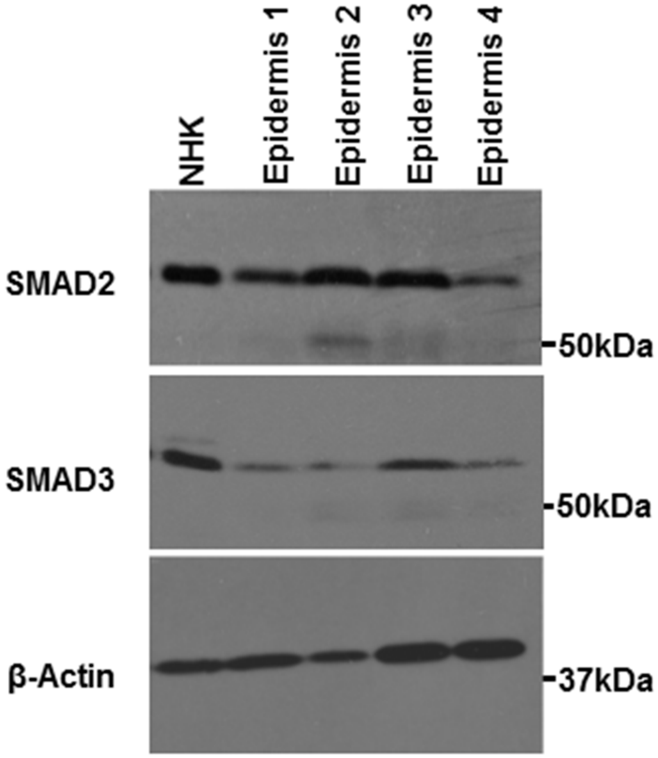

Supplementary Figure 2: Analysis of SMAD2 and SMAD3 protein in normal human keratinocytes. Protein extracts from normal human keratinocytes (NHK) and normal skin epidermis from four donors were analysed by SDS-PAGE and western blotting for total SMAD2 and SMAD3. β-Actin was included as a loading control.

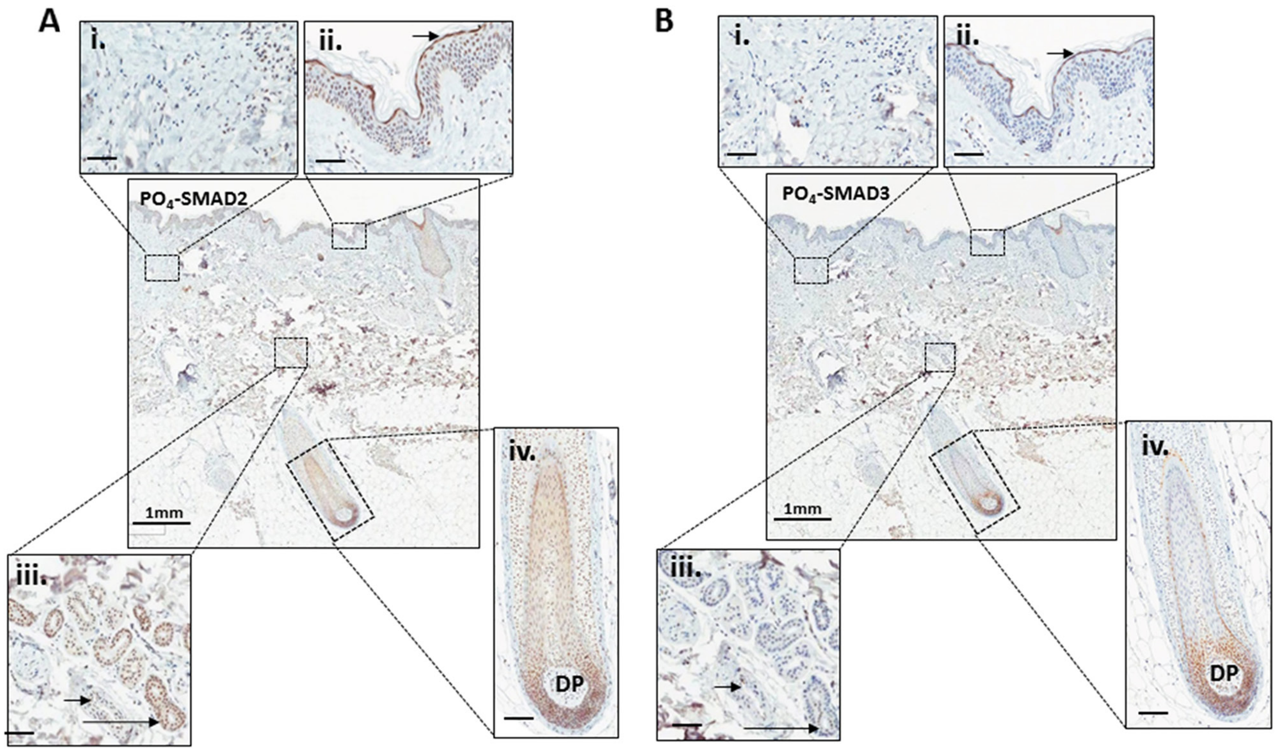

Supplementary Figure 3: Distribution of positive nuclear anti-PO_4-SMAD immuno-reactivity in normal human scalp skin. **(A)** PO_4-SMAD2: Main central image - IHC of normal scalp skin stained with anti-PO_4-SMAD2 antibody at x4 magnification. Separate boxes display sites of positive staining expanded to x10 magnification including; **i.** Dermis **ii.** Epidermis (Arrow: non-specific staining of kerato-hyaline in granular layer **iii.** Eccrine Glands (Long arrow) and blood vessels (Short arrow) **iv.** Hair follicle bulb and dermal papilla (DP). Scale bars = 300µm. **(B)** PO_4-SMAD3: Main central image - IHC of normal scalp skin stained with anti-PO_4-SMAD3 antibody at x4 magnification. Separate boxes display sites of positive staining expanded to x10 magnification including; **i.** Dermis **ii.** Epidermis (Arrow: non-specific staining of kerato-hyaline in granular layer **iii.** Eccrine Glands (Long arrow) and blood vessels (Short arrow) **iv.** Hair follicle bulb and dermal papilla (DP). Scale bars = 300µm.

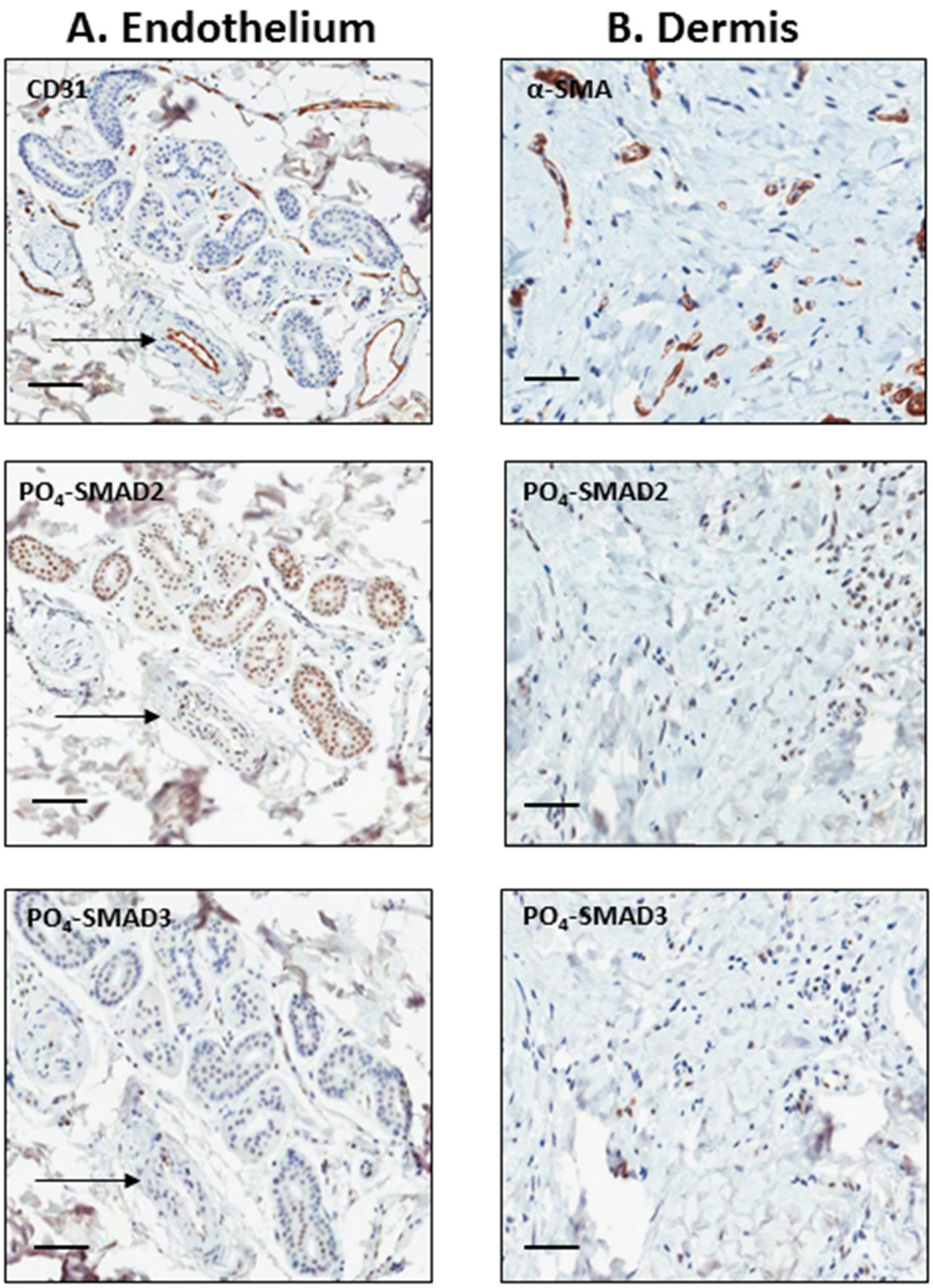

Supplementary Figure 4: Endogenous TGF-β signalling within blood vessel walls. Representative images of normal scalp skin IHC stained with: **(A)** Endothelial cell marker CD31 and **(B)** Dermal fibroblast/myofibroblast marker αSMA. Corresponding serial sections stained with both PO_4-SMAD2 and PO_4-SMAD3 below. Scale bars = 300µm.

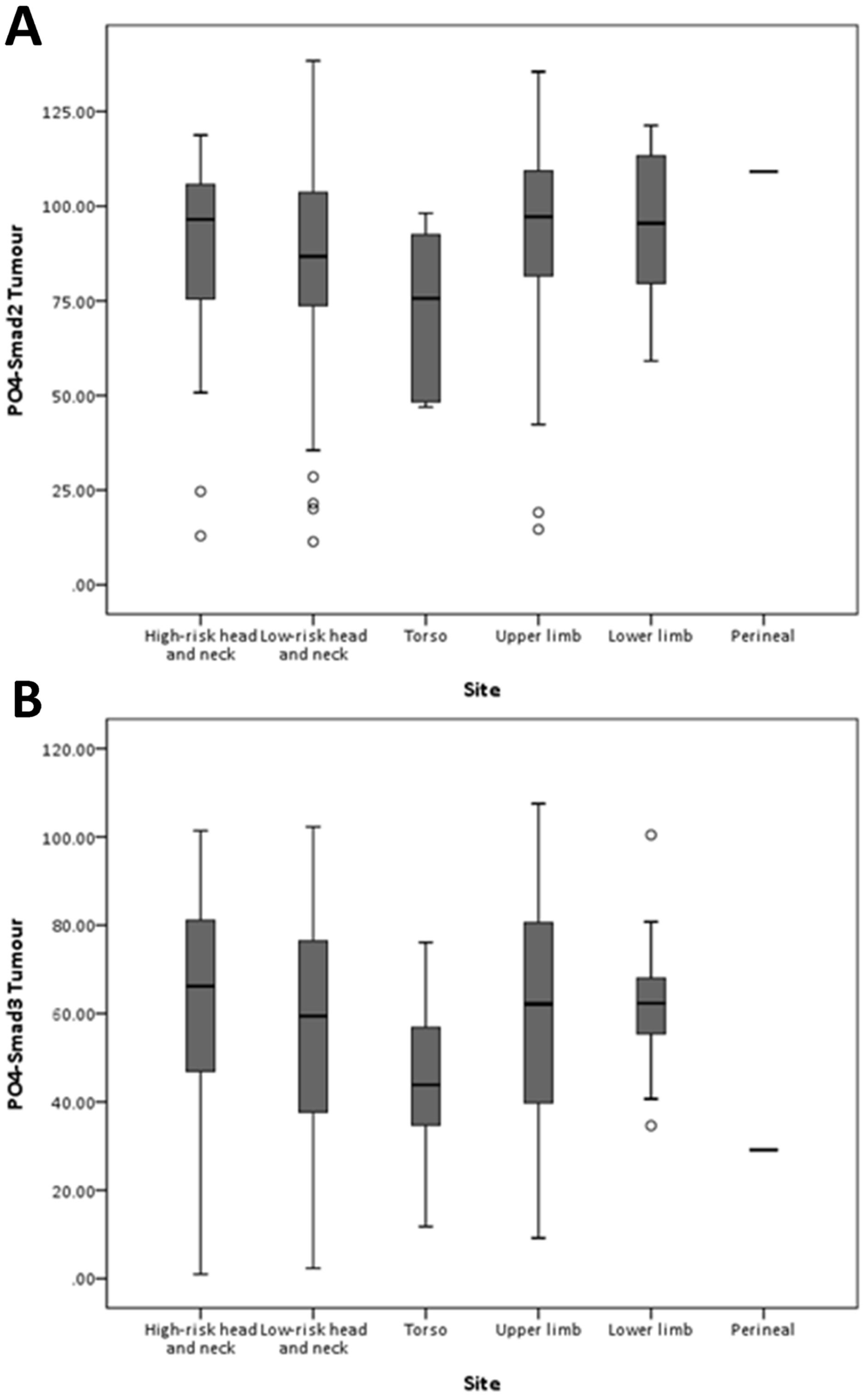

Supplementary Figure 5: TMA histoscores identify no significant difference between mean nuclear R-SMAD expression and anatomical site of cSCC. (A) NuclearPO_4-SMAD2 activity by cSCC site as labelled. **(B)** Nuclear PO_4-SMAD3 activity by cSCC site as labelled. High-risk head and neck defined as ear and external lip. Analysis of Variance (ANOVA) Sig * p = <0.05, ** p = <0.01.

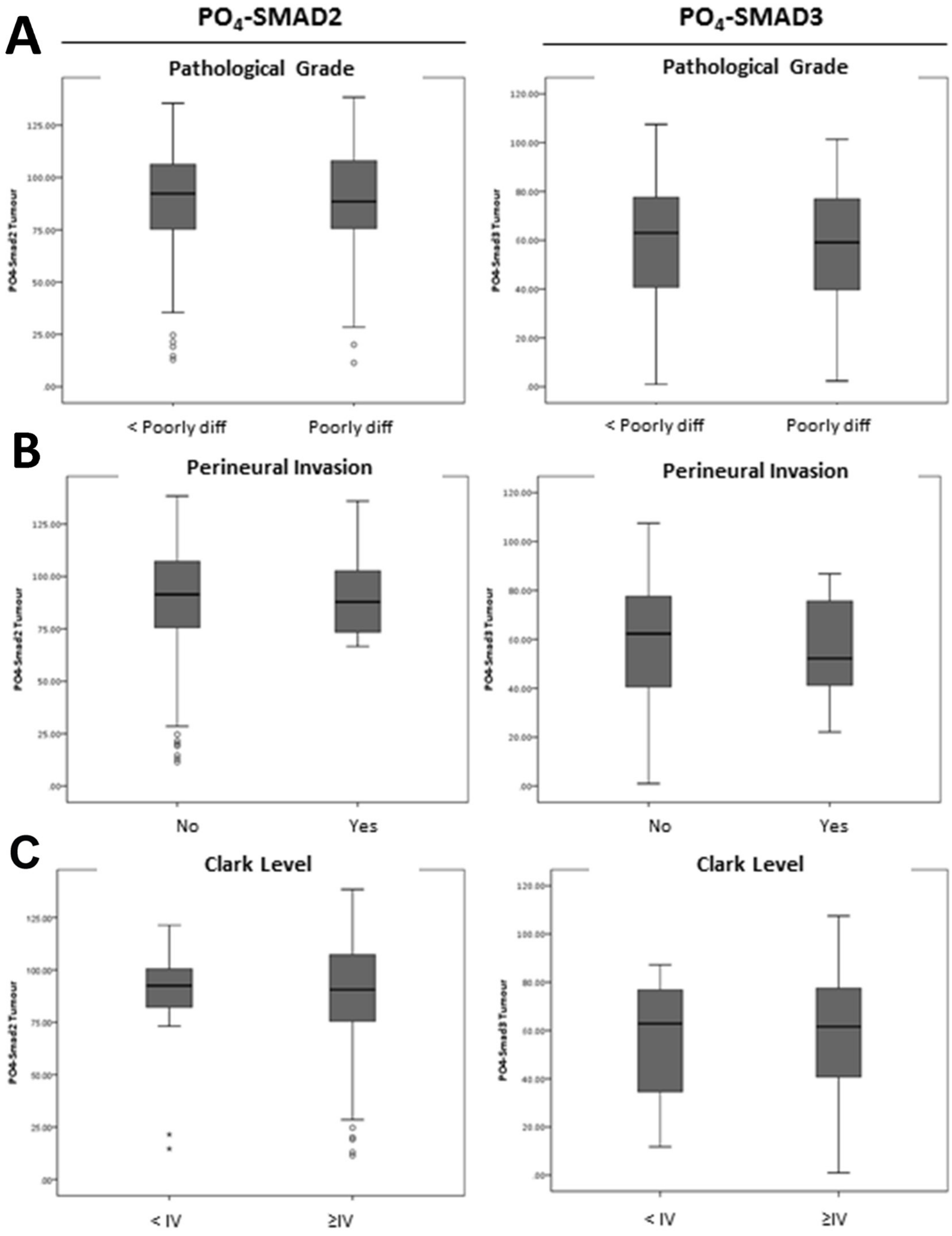

Supplementary Figure 6: TMA histoscores identify no significant association between mean nuclear R-SMAD expression and cSCC grade, perineural invasion and Clark level. **(A)** Nuclear PO_4-SMAD2 and PO_4-SMAD3 activity by pathological grade (Defined as < Poorly differentiated or Poorly differentiated) **(B)** Nuclear PO_4-SMAD2 and PO_4-SMAD3 activity by perineural invasion (Defined as No or Yes) **(C)** Nuclear PO_4-SMAD2 and PO_4-SMAD3 activity by Clark level. (Defined as <IV (Invasion superficial to reticular dermis) or ≥IV (Invasion at or deep to reticular dermis). Analysis of Variance (ANOVA) Sig * p = <0.05, ** p = <0.01.

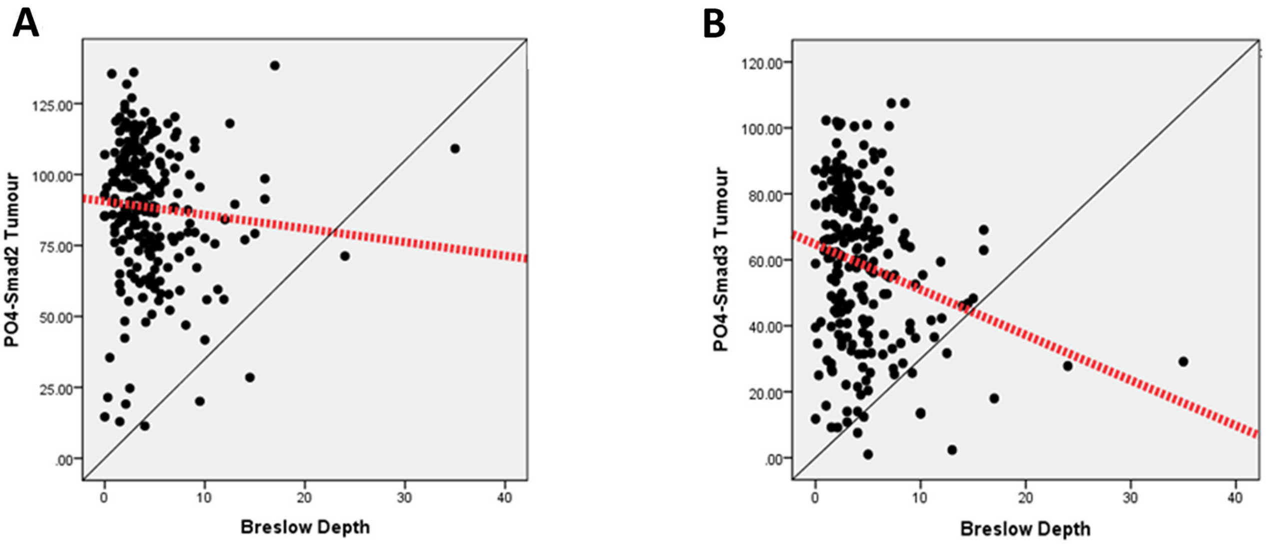

Supplementary Figure 7: Scatterplots of TMA histoscores versus tumour Breslow depth for PO_4-SMAD2 and PO_4-SMAD3. **(A)** Nuclear PO_4-SMAD2 activity by tumour Breslow depth. Red dot line = Linear best fit. $R^2 = 0.006$ **(B)** PO_4-SMAD3 activity by tumour Breslow depth. Red dot line = Linear best fit. $R^2 = 0.053$.
